# Understanding factors influencing personal care workers' intentions to stay: A systematic integrative review

**DOI:** 10.1111/ajag.70017

**Published:** 2025-03-17

**Authors:** Britt O'Keefe, Eva Yuen, Susan Perlen, Briony Dow, Alison M. Hutchinson

**Affiliations:** ^1^ School of Nursing and Midwifery: Center for Quality and Patient Safety Research Deakin University Geelong Australia; ^2^ Monash Health Clayton Victoria Australia; ^3^ Barwon Health Geelong Victoria Australia; ^4^ National Ageing Research Institute Inc Melbourne Victoria Australia; ^5^ Deakin University School of Nursing and Midwifery Melbourne Victoria Australia; ^6^ School of Population and Global Health University of Melbourne Melbourne Victoria Australia; ^7^ Faculty of Health Sciences University of Southern Denmark Odense Denmark

**Keywords:** caregivers, homes for the aged, retention

## Abstract

**Objective:**

To systematically synthesise existing literature to identify influences on personal care workers' intentions to stay when employed in residential aged care.

**Methods:**

A systematic integrative review was conducted, searching relevant literature across multiple databases, including Business Source Complete, CINAHL Complete, Medline Complete and APA PsycInfo via the EBSCOhost platform and EMBASE (excluding Medline). Articles were eligible for inclusion if they were peer‐reviewed, published in English between 1997 and 2024, and focused on factors influencing personal care workers' intentions to stay in residential aged care. The methodological quality of the selected articles was assessed using the Mixed Methods Appraisal Tool.

**Results:**

This review included six articles published between 2007 and 2021, reporting on quantitative (*n* = 4), qualitative (*n* = 1) and mixed methods (*n* = 1) research, with the final study published in 2021. Five employee‐related and employer‐related themes emerged as important factors that influence personal care workers' intentions to stay: (1) individual characteristics and resilience, (2) career growth and rewards, (3) employment stability and economic workforce pressures, (4) satisfaction and fulfilment in professional caregiving and (5) organisational support and collaborative work environment.

**Conclusions:**

This review identified key factors influencing personal care workers' intentions to stay, offering actionable insights to inform the development of evidence‐based strategies aimed at strengthening workforce retention. Addressing these factors is critical to meeting the needs of an ageing population and ensuring the provision of high‐quality care. Furthermore, the review underscores the multifaceted determinants shaping these intentions, emphasising the importance of psychological, social and organisational factors to enhance retention efforts.

**Practice impact**

This review highlights key individual characteristics and organisational factors influencing personal care workers' employment intentions, offering actionable insights for residential aged care operators, policymakers, and researchers. Collaboratively addressing these factors enables the development of evidence‐based strategies promoting employee retention, ensuring stability while ultimately improving the quality of care for residents.

## 1 INTRODUCTION

The global ageing phenomenon is associated with a rapid rise in the number of individuals living longer.^1^ By 2050, the population aged 60 years and over is estimated to reach approximately 2.1 billion, while the number of individuals aged 80 years and older is projected to increase nearly threefold to 425 million by 2050.^2^ Australia is facing similar projected population challenges. In 2020, an estimated 4.1 million Australians, constituting 16% of the total population, were aged 65 years or older.^3^ This percentage is projected to grow and represent about a quarter of the total population by 2071.^4^ It is anticipated that these demographic projections will place significant pressure on the aged care sector.

Globally, residential aged care (RACs) is referred to by various terms, such as nursing homes, long‐term care homes and care homes. Despite differing terminology, they share commonalities of providing medical and non‐medical care and support 24 h a day, 7 days a week for older people unable to live independently.^5^, ^6^, ^7^ In 2024, approximately 200,000 older Australians received supports and services in an RAC setting, an increase of approximately 5300 residents compared with the previous year.^8^ This upward trend reflects the growing demand for RAC services, driven by an ageing population. In 2020, RAC operators employed nearly 209,000 direct care employees, comprising nurses (23%), personal care workers (PCW) (70%) and allied health professionals (7%), to care for RAC residents as a core component of their work.^9^ This workforce composition underscores the critical contribution of the PCW role in delivering and maintaining high‐quality care for RAC residents.

As the largest segment of the direct care workforce in RACs,^9^, ^10^ PCWs provide most of the personal, physical and emotional support to aged care residents, assisting with activities of daily living ranging from toileting, showering and dressing to eating and assistance with social activities.^11^ Often regarded as the ‘backbone’ of the RAC workforce,^12^ globally, PCWs are identified by a variety of titles such as certified nursing assistant, personal support worker and care assistant. Despite the key role PCWs play in providing day‐to‐day care for RAC residents, over 12 months from November 2020, 28% of PCWs in Australia left their employment, and almost half of RAC operators reported at least one vacant PCW position, with an average of five vacancies per facility.^10^ While some degree of workforce turnover can bring innovation and new perspectives, not all turnover is deemed undesirable^13^; a turnover rate of 28% poses significant challenges for RAC operators, such as financial strain^14^ and potential adverse outcomes for residents, including a decline in care quality.^6^ High turnover underscores the workforce challenges facing RAC operators, undermining care continuity and quality. A review of the existing literature indicates that turnover among PCWs is associated with a higher incidence of falls, pressure ulcers, contractures, catheter use, greater use of physical restraint, higher hospital admission rates and an increased mortality rate among RAC residents.^15^, ^16^, ^17^

‘Intention to stay’ (ITS) refers to an employee's intent to remain with their current employer,^18^ while ‘retention’ refers to the extent to which staff remain employed with the current employer.^19^ Intention to stay is often considered a precursor or predictor of retention, with ITS being used as a proxy for retention.^20^, ^21^, ^22^, ^23^ While ITS is a critical indicator of employee retention,^24^ researchers have identified few factors that influence both employees' ITS and employee retention.^24^, ^25^, ^26^ A study of 595 nursing assistants found that among 18 variables, only emotional exhaustion significantly impacted ITS and retention.^24^ Similarly, Dill et al.^25^ observed that being the primary breadwinner was the sole factor significantly associated with both employment intentions to remain in one's current position and employee retention in a study of 315 nursing assistants. Howe et al.^26^ argued that the factors driving ITS are not merely the inverse of those influencing retention. This distinction is crucial, as it indicates that separate factors influence workers' ITS and actual retention. Understanding this distinction enables RAC operators, policymakers and researchers to address the underlying drivers of turnover while promoting employee retention. By employing evidence‐based strategies that are specifically tailored to the unique factors influencing an employee's ITS, separate from strategies used to enhance aimed at promoting retention, targeted interventions can be developed.

Over the past four decades, researchers have extensively investigated the employment intentions of nurses in hospital and nursing home settings to comprehensively understand factors associated with nurses' employment intentions and actual turnover.^27^, ^28^, ^29^, ^30^ Given the very limited number of reviews examining the factors influencing PCWs' ITS, the objective of this research was to systematically synthesise existing literature to identify factors that influence PCWs' ITS. The research question posed was as follows: What factors influence intention to stay among personal care workers employed in residential aged care? Understanding these factors is the crucial first step towards developing evidence‐based strategies to promote the retention of PCWs, the largest segment of the direct care workforce in RAC. Retaining PCWs is essential for ensuring the continuity of personalised, high‐quality care to meet the needs of an ageing population.^31^

## 2 METHODS

The systematic search strategy incorporated three dimensions: ITS, intention to leave and actual turnover among PCWs employed in a RAC setting. The protocol included a research question for each dimension and was prospectively registered on PROSPERO. Data from relevant literature regarding each dimension underwent independent analysis. This paper reports the findings related to the ITS dimension, while findings for intention to leave are under review and a manuscript for actual turnover is in preparation.

### 2.1 Design

An integrative review approach was selected for this study, incorporating quantitative, qualitative and mixed methods methodologies, to provide a comprehensive understanding of the factors influencing PCWs' ITS.^32^ Whittemore and Knafl's^32^ methodological framework was used to guide the review, and reporting adheres to the Preferred Reporting Items for Systematic reviews and Meta‐Analysis (PRISMA) guidelines.^33^, ^34^ This approach allows for the integration of existing literature, offering insights into current research trends^35^ and the identification of future research paths, whilst informing the application of findings for both practical use and policy development.^32^

### 2.2 Search strategy

A comprehensive search was conducted in July 2022 and updated in March 2024 across multiple databases, including Business Source Complete, CINAHL Complete, Medline Complete, and APA PsycInfo via the EBSCOhost platform and EMBASE (excluding Medline). The search strategy was developed in consultation with the research librarian and included the following terms and their synonyms: personal care worker, residential aged care, ITS, intention to leave and actual turnover. The terms and concepts were combined using Boolean operators ‘AND’ and ‘OR’ (Appendix S1). Additional eligible articles were identified by systematically hand‐searching the reference lists of the included articles.

### 2.3 Inclusion and exclusion criteria

Articles that met the eligibility criteria (Table 1) were included. Studies involving participants other than PCWs or from other aged care environments were included if the authors reported separate results for PCWs in RAC. Literature reviews (systematic, integrative, scoping, rapid and umbrella reviews), conference proceedings, abstracts, dissertations, protocols, letters, editorials, commentaries, books and grey literature were excluded.

**TABLE 1:** Eligibility criteria for articles included in this review.

Criteria	Eligibility requirements
Publication year	Articles published after 1 July 1997, aligning with the marketisation of Australia's aged care sector initiated by the *Aged Care Act 1997*, which introduced private RAC providers, were included
Article type	Peer‐reviewed, English‐language articles reporting empirical studies
Study design	Articles reporting quantitative, qualitative, or mixed methods studies, with no restriction on study design
Population	PCWs (also known as certified nursing assistants, nursing aides or care assistants)
Setting	Studies conducted in RAC (also known as nursing homes, long‐term care homes and care homes)
Dimension	Intention to stay

### 2.4 Article screening

**FIGURE 1:**
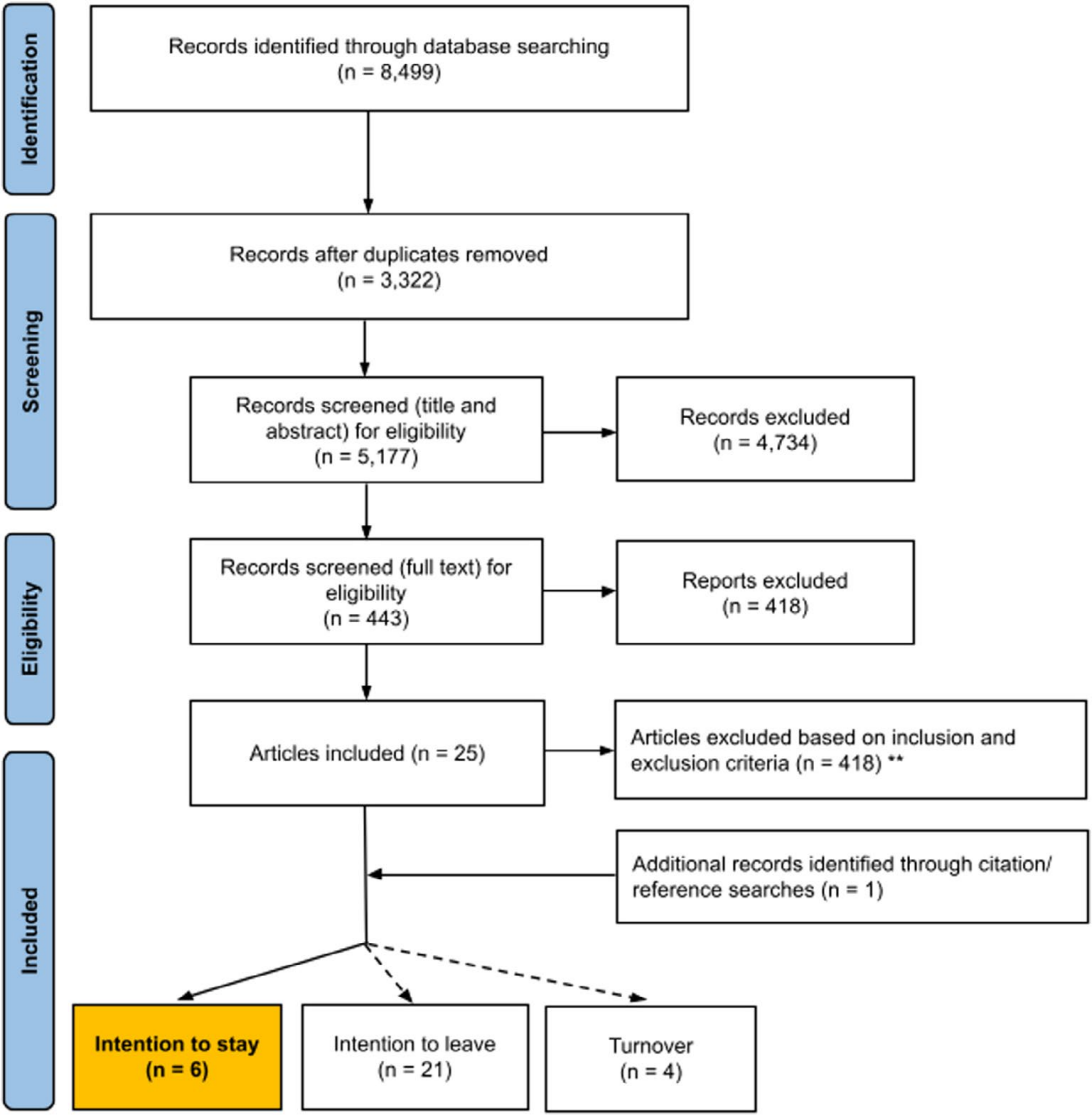
PRISMA flowchart diagram*. *Of the 26 articles identified, some reported more than one of the concepts in a single study. As a result, some articles are included in multiple categories. **Articles (*n* = 418) were excluded for the following reasons: Not about the dimension (*n* = 98), Proxy respondent (completed by non‐PCWs on PCWs' behalf) (*n* = 82), Results—no differentiation between roles (*n* = 78), Not research (*n* = 75), Completed by non‐PCW staff (*n* = 34), Setting—not residential aged care (*n* = 27), Results—no differentiation between settings (*n* = 17), Results—no differentiation between job types (*n* = 5), and Abstract only (*n* = 2).

Articles were imported into EndNote, duplicates were automatically removed, and the remaining articles were imported into Covidence for screening.^36^ A two‐stage screening process was conducted, with two authors independently screening articles at each stage. In the first stage, publication titles and abstracts were screened. In the second stage, full‐text articles were screened. Discrepancies were resolved through verbal discussion to reach a consensus. Reasons for excluding full‐text articles are shown in Figure 1.

### 2.5 Quality appraisal

The authors used the Mixed Methods Appraisal Tool (MMAT) version 2018,^37^ to assess the methodological quality of the included articles. Two authors independently assessed all studies and resolved disagreements through verbal discussion. The methodological quality was scored on a scale of 1 to 5, where 1 indicated poor quality and 5 indicated excellent quality. Employment of the MMAT facilitated the identification of methodological strengths and weaknesses in the studies included in this review.

### 2.6 Data extraction and analysis

Following Whittemore's framework involving data reduction, data display, data comparison, conclusion drawing and verification,^32^ a predefined data extraction table was developed and used to extract data from each study. For quantitative studies, where bivariate and multivariate analyses were reported, only the statistically significant variables from the most robust level of analysis were extracted (Appendix S2); non‐statistically significant variables from the included quantitative studies are reported in Appendix S3. Qualitative data were extracted and analysed thematically to identify patterns and generate themes (Appendix S4).^38^ An iterative process was used to integrate quantitative and qualitative data, identifying themes.^32^ One author extracted all data, which were independently reviewed by another author for accuracy against the primary source.^39^ Data were cross‐verified by two authors against the primary source.^39^

## 3 RESULTS

### 3.1 Search results

After removing duplicates, 5178 articles underwent title and abstract screening, resulting in the exclusion of 4734 articles, and 444 articles remained for full‐text review. A further 418 articles were excluded during the full‐text review, resulting in a final set of 25 articles investigating ITS, intention to leave and/or turnover. One additional study was found through hand‐searching reference lists of included articles. Of the 26 articles, six articles (highlighted in Figure 1) specifically focused on ITS and were included in the current review.

### 3.2 Study characteristics

Intention to stay study findings were reported in six articles over 24 years. Of the six included articles, the first was published in 2007, followed by a second article in 2008. Additional articles were published in 2013 and2015. After another 5‐year hiatus, further articles were published in 2020, with the final study included in this review published in 2021. The studies were conducted in the United States (*n* = 3), Australia (*n* = 2) and Taiwan (*n* = 1). Study designs included cross‐sectional (*n* = 4), qualitative (*n* = 1) and mixed methods design (*n* = 1) (Table 2). Sample sizes ranged from 16 to 333 across 1 to 41 RAC facilities. Additional information is provided in Appendices S2 and S4.

**TABLE 2:** Summary of included studies.

Author, (year), country	Aim/objective/purpose	Design	Sample and setting	Findings/Themes
Bishop et al.,^20^ (2008), USA	To investigate (a) whether CNAs are more committed to NH jobs when they perceive their jobs as enhanced (greater autonomy, use of knowledge, teamwork), and (b) whether CNA job commitment affects resident satisfaction	Cross‐sectional, surveys	*n* = 255 255 CNAs from 18 NHs	*Individual* Age >45 years (+) *Organisational* Employee benefits (+) Career advancement (+) Basic supervision (+)
Chang et al.,^40^ (2021), Taiwan	To investigate the influences of NAs job competency on their intrinsic and extrinsic satisfaction and their ITS in the profession of LTC institutions	Cross‐sectional questionnaire	*n* = 333 333 NAs from 26 NHs and 15 elderly welfare institutes	*Organisational* Intrinsic satisfaction (+) Extrinsic satisfaction (+) Job competency → intrinsic satisfaction (+) Job competency → extrinsic satisfaction (+) Job competency → extrinsic satisfaction → intrinsic satisfaction (+)
Dhakal et al.,^31^ (2020), Australia	To explore the attraction and retention of ACAs and to identify specific ‘push‐and‐pull’ factors influencing the intention of ACAs to stay in their current job or leave for other occupations	Cross‐sectional survey	*n* = 79 79 ACAs from 9 RAC facilities	*Individual* Aged >40 years (+) Education attainment of degree/diploma or higher (+) Language spoken at home other than English (+) Permanent or temporary employment status (+) *Other* Non‐metropolitan location (+)
Dill et al.,^25^ (2013), USA	To examine the relationship between job satisfaction, ITS, and retention among low‐wage health‐care workers, specifically focusing on NAs in NHs	Cross‐sectional survey	*n* = 315 315 NAs from 18 NHs	*Individual* Breadwinner (+) *Organisational* Supervisor support (−) Financial rewards (+) Career rewards (+) Perceived quality of care (+) *Other* Public assistance (+)
Gao et al.,^41^ (2015), Australia	To understand individual DCWs' perceptions of the rewards and difficulties of RAC work, how these were related to their employment intentions, and how these varied between nurses and NAs, and the cultural diversity of workers	Individual semi‐structured interviews	*n* = 16 16 NAs from 1 RAC facility	*Individual* Employment characteristics Meaning of care work *Organisational* Nature of care work Organisational resources
Yeatts and Cready,^42^ (2007), USA	To evaluate the effects of empowered work teams, specifically designed to empower CNAs, within the LTC setting	Mixed methods, observations, empowered work team weekly summaries, and nurse management weekly written responses	*n* = 314–353 314–353 CNAs from 10 NHs	*Organisational* Empowered CNA teams

*Note*: + = positive association with ITS; − = negative association with ITS; Sample size = the respective study's sample for analysis. The findings from the qualitative study were extracted from the study prior to being thematically analysed.

Abbreviations: ACA, aged care assistant; CNA, certified nursing assistant; DCW, direct care worker; ITS, intention to stay; LTC, long‐term care; NA, nursing assistant; NH, nursing home; RAC, residential aged care; RAC, residential aged care facility; SS, sample size; USA, United States of America.

### 3.3 Methodological quality of included studies

Using the MMAT, four of the six studies (three quantitative and one qualitative) were scored as excellent quality, while two were assessed as good quality (a quantitative and a mixed methods study) (Appendix S5). Unclear reporting on non‐response bias, insufficient rationale for the selection of statistical analyses in the quantitative descriptive study, and inadequate justification for the chosen mixed methods design resulted in lower quality ratings of good quality for two studies. Additionally, the quantitative and qualitative components of the mixed methods study did not fully adhere to the respective quality criteria and were therefore assessed as good quality. Despite these methodological weaknesses, all studies were retained for review.

### 3.4 Narrative synthesis of results

Synthesis of the findings of the six included studies revealed five themes influencing PCWs' ITS: (1) individual characteristics and resilience, (2) career growth and rewards, (3) employment stability and economic workforce pressures, (4) satisfaction and fulfilment in professional caregiving and (5) organisational support and a collaborative work environment.

#### 3.4.1 Individual characteristics and resilience

Age was associated with PCWs' ITS. Employees over 40 years^31^ and over 45 years of age^20^ demonstrated a stronger desire to stay in their current roles compared to younger colleagues. Additionally, higher education attainment was associated with higher ITS. Employees who spoke a language other than English demonstrated lower ITS compared to those who spoke English at home.^31^ Job resilience was also positively associated with increased ITS.^41^

#### 3.4.2 Career growth and rewards

Greater satisfaction with career advancement opportunities^20^ and career rewards, including promotions,^25^ was associated with increased ITS. Additionally, higher satisfaction with employee benefits^20^ and financial rewards^25^ was also associated to increase ITS.

#### 3.4.3 Employment stability and economic workforce pressures

Permanent or temporary employment, rather than casual contracts,^31^ and being the primary breadwinner^25^ were associated with increased ITS. Additionally, job and workplace security, retention of older workers and promoting employee equity^41^ were associated with increased ITS. Despite low remuneration, some employees' negative perceptions were negated by their professional fulfillment.^41^

Receiving public assistance, such as welfare payments or food stamps,^25^ and workplace location^31^ were also associated with ITS. Specifically, employees receiving public assistance in the past three years^25^ and those working in non‐metropolitan areas exhibit higher ITS.^31^

#### 3.4.4 Satisfaction and fulfilment in professional caregiving

Extrinsic satisfaction was derived from external factors such as salary, benefits, promotions, working conditions, relationships with colleagues and supervisor support. Intrinsic satisfaction was derived from the actual work and the internal rewards it provided, such as a sense of achievement, responsibility, recognition, personal growth and the meaningfulness of work tasks. Both extrinsic and intrinsic satisfaction contributed to higher ITS.^40^ A nuanced relationship between job competency and ITS, mediated by extrinsic and intrinsic satisfaction, was reported, with job competency found to influence extrinsic satisfaction, which in turn influenced intrinsic satisfaction, ultimately positively impacting ITS.^40^

Having an emotional connection with residents and the perceived standard of care was associated with an employee's ITS.^41^ In particular, employees' perceptions of high quality of care in their current workplace were strongly associated with a higher ITS.^25^

#### 3.4.5 Organisational support and collaborative work environment

Organisations that promoted workplace health and safety, provided access to organisational resources, with supportive managers,^41^ offered basic supervision pertaining to a foundational level of oversight and support provided by supervisors^20^ and supervisory support,^25^ were associated with higher ITS. Additionally, empowered teams^42^ and effective teamwork^41^ were also positively associated with ITS.

## 4 DISCUSSION

This review included six articles, each reporting separate studies that identified factors influencing PCWs' ITS.^20^, ^25^, ^31^, ^40^, ^41^, ^42^ Four of the six studies utilised cross‐sectional designs to examine ITS based on predetermined variables.^20^, ^25^, ^31^, ^40^ Despite the scarcity of evidence in this review, the included studies identified key factors influencing ITS among PCWs.

The review revealed that sociodemographic characteristics, including language spoken at home, were associated with PCWs' ITS. Personal care workers aged 40 years and older demonstrated a stronger desire to stay in their current roles than their younger colleagues,^20^, ^31^ aligning with findings from Choi and Johantgen^43^ and Sloane et al.,^44^ who reported that older PCWs were more likely to stay. This may be attributed to older employees' greater investment in residents, having established roles within the organisation, and higher job stability and satisfaction. Employees who spoke a language other than English at home were less likely to stay,^31^ potentially due to language barriers, cultural differences or workplace isolation that may affect job satisfaction and retention among culturally diverse PCWs, has been found to be significant.^45^ Given the large proportion of culturally and linguistically diverse PCWs,^10^ particularly immigrants working below their level of qualifications from their home country,^45^, ^46^ there is a need to understand further the factors that influence ITS among this population.

Previous researchers have frequently considered ITS as a precursor or predictor of retention, often using ITS as a proxy for retention.^20^, ^22^, ^23^ Developed by Ajzen,^47^ the Theory of Planned Behavior (TPB) provides a robust framework for predicting and explaining human behaviour, including employee intentions.^47^ While widely applied across fields such as occupational health, management and hospitality,^48^ it has seldom been used to explore direct care workers' behavioural intentions to stay in their role. The TPB assumes that behavioural intentions, shaped by attitudes, subjective norms and perceived behavioural control, are strong predictors of actual behaviour, which can offer valuable insights into understanding and predicting employee behaviour.^47^

This review demonstrated that the three determinants of behavioural intentions, attitudes, subjective norms and perceived behavioural control,^47^ were found to influence PCWs' employment intentions. Interpersonal influences, including supportive management and team dynamics influenced subjective norms by fostering a shared understanding of expectations, creating a sense of collective responsibility and reinforcing social pressures to align with organisational values. While resource availability, job security and factors affecting PCWs' sense of control shaped their perceived behavioural control, influencing their confidence in performing their role effectively. Beyond these characteristics, this review revealed statistically significant factors such as job satisfaction, rewards and workplace conditions shaping PCWs' attitudes by influencing their perception and emotional connection to their roles.

Job satisfaction is frequently identified as a prominent factor influencing PCWs' ITS.^49^, ^50^ In a study of Dutch naval professionals using the three determinants of TPB along with job satisfaction and other variables such as age, tenure and commitment, Van Breukelen et al.^51^ concluded that TPB significantly predicted turnover intentions, suggesting that both attitudes and subjective norms significantly predicted turnover intentions. In this review, only one study examined job satisfaction, reporting an interrelationship between intrinsic and extrinsic job satisfaction job competency and ITS, highlighted the importance of strategies to promote employees' competence and job satisfaction to influence their ITS.^40^ Defined as the ability to perform tasks requiring specific knowledge or skills,^52^ job competency was revealed as a key determinant in shaping the employment intentions of aged care workers. Austen et al.^53^ found that higher work ability, closely tied to job competency, was strongly correlated with a greater likelihood of remaining in one's position. In contrast, insufficient job competency could reduce ITS by lowering self‐esteem and increasing workplace stress as employees struggled to manage and complete tasks effectively.^54^ These findings highlighted the need for RAC operators to invest in competency development programs, targeting skill development to increase job competency, leading to higher job satisfaction and improved retention among PCWS in the aged care sector.

The findings of this review underscore the key organisational attributes, including supportive management,^25^, ^41^ job security^41^ and a safe working environment,^41^ in enhancing PCWs' ITS. Our findings support existing studies,^55^, ^56^ which found that organisational leadership, which supports the emotional and mental well‐being of PCWs through respectful and supportive supervisory practices, influences ITS. In addition, addressing the financial challenges faced by many PCWs, offering competitive salaries with secure permanent or temporary contracts was found to influence ITS,^31^ since it reduces reliance on casual employment and contributes to job stability. Moreover, ensuring a safe working environment with manageable workloads is vital for maintaining high‐quality care and cultivating a positive organisational culture that encourages PCWs to remain in their roles.^50^ According to the TPB, together, these factors may influence PCW's attitudes towards the role; PCWs who feel supported by their managers and valued by the organisation are likely to develop positive attitudes towards their role, thereby increasing their intentions to stay in the position.

Empowered teams^42^ and effective teamwork^41^ were linked to higher ITS. In the busy and demanding environment of aged care, researchers found that PCWs valued autonomy and decision‐making authority to respond promptly and appropriately to residents' care needs.^57^, ^58^ While responding to supervision, PCWs need to be empowered to not only advocate for aged care residents but also make decisions and take actions within the scope of their job role and responsibilities, ultimately increasing employees' ITS.^59^, ^60^ In the context of TPB, a workplace environment which empowers PCWs and fosters effective teams likely influences their subjective norms of the role; by engaging PCWs through supporting and valuing their vital role in caring for older adults, it reframes the perception of their work from ‘dirty work’ to a respected role, which can influence their ITS.^61^, ^62^, ^63^

Additionally, career advancement^20^, ^25^ and career rewards in the form of benefits, recognition and opportunities^25^ influenced PCWs' ITS. Career advancement opportunities and rewards in organisations that prioritise employee growth and provide equitable pathways for advancement shape PCWs' attitudes towards the role. According to TPB, these factors shape PCWs' perceptions of future prospects, enhancing their job satisfaction, motivation and commitment, strengthening their intentions to stay. However, with poorly defined career pathways for PCWs, the aged care sector has been viewed as a stepping stone for career progression into higher status roles.^64^, ^65^, ^66^, ^67^ To maintain a strong and stable personal care workforce, clearly defined career pathways and structured advancement opportunities within the sector are crucial for maintaining employee motivation and commitment.

Higher compensation and benefits were found to shape PCWs' intentions to stay. However, Gao et al.^41^ noted that some employees offset negative perceptions of insufficient income with professional fulfilment, suggesting that financial compensation alone is not sufficient to retain workers. Gyllensten et al.'s^66^ study of older assistant nurses revealed the significance of employees' roles in caring for aged care residents, perceiving the work as deeply meaningful. An emotional connection with residents and a perceived higher standard of care quality significantly influenced employees' ITS.^25^ These findings align with those of Senecal et al.,^14^ who found that positive caring relationships and ensuring a professional commitment to residents' happiness and well‐being were central to the nursing assistant's role. The authors noted that nursing assistants felt most fulfilled when providing high‐quality, person‐centred care.^14^ Fostering opportunities to enhance emotional connections between PCWs and residents, as well as optimising quality of care, has the potential to increase PCWs' ITS.

This review showed that receiving public assistance, such as welfare payments or food stamps^25^ and working in non‐metropolitan areas,^31^ significantly influenced employees' ITS. Receiving public assistance, may, according to TBP, enhance perceived behavioural control through financial stability, while working in non‐metropolitan areas may foster employee intentions to stay by reinforcing norms of loyalty and community among PCWs. Choi and Johantgen's^43^ analysis of the 2004 National Nursing Home Survey and the 2004 National Nursing Assistant Survey data involving 2254 nursing assistants found that those working in metropolitan areas had a higher intention to leave compared to those in micropolitan or rural areas. Metropolitan areas presented challenges such as higher living costs, longer commutes and reduced work‐life balance, affecting employees' intentions to stay. Wheatley^68^ studied 5336 individuals from dual‐career households and found the physical workplace location was particularly crucial for female employees' ITS, given their personal responsibilities. This is particularly important as approximately 86% of the RAC direct care workforce, including nurses, PCWs and allied health professionals, are women,^10^ and therefore, the physical workplace location may play a pivotal role in PCWs' ITS. Addressing the increasing shortage of skilled workers in the RAC sector is critical. However, as evidenced by this review, the limited number of studies examining ITS among PCWs means a large gap exists in understanding this complex issue.

In the context of employee retention, applying TPB requires RAC operators to consider the complex and dynamic interplay of psychological, social and organisational factors shaping the employment intentions of PCWs. Through improved levels of job satisfaction, enhanced interpersonal support and availability of resources, RAC operators, policymakers and researchers can use the three determinants of TPB strategies to develop evidence‐based strategies that promote retention and ultimately improve the quality of care for residents.

### 4.1 Strengths and limitations

The strengths of this review include the use of a rigorous, systematic and reproducible method and the inclusion of research studies that incorporated various research methodologies. However, the review had several limitations. Despite a comprehensive search, the small number of studies included may mean that the full range of factors related to ITS among PCWs in RAC facilities may not have been identified. Additionally, the studies included in this review were conducted in three countries, limiting the generalisability of the findings. Only studies published in English in peer‐reviewed journals were included, meaning potentially relevant articles may have been excluded. Most studies used cross‐sectional designs, restricting the ability to draw causal conclusions between preselected variables and ITS. We did not contact the study authors to clarify ambiguous details, which could have affected the quality assessment.

## 5 CONCLUSIONS

The findings of this review provide valuable insights for RAC operators, policymakers and researchers to develop evidence‐based strategies that promote the retention of PCWs. Addressing both employee and organisational characteristics influencing PCWs' ITS offers a promising avenue for retaining a strong and stable workforce to ultimately improve the quality of care for residents. Such efforts are essential to meet the evolving needs of the ageing population and ensure a sustainable workforce in aged care.

To build on the limited evidence in this area, future research is needed to investigate how changes in, and interactions between the individual and the organisation influence PCWs' ITS. Additionally, further qualitative research that explores both current and former PCWs' experiences and perceptions that influence ITS could provide much‐needed evidence for a deeper understanding of the issues and potential opportunities for informing the development, implementation and evaluation of targeted strategies to retain PCWs in the workforce.

## FUNDING INFORMATION

Britt O'Keefe is supported by a Postgraduate Research Scholarship from Deakin University. The researchers did not receive any specific grants from any funding agency in the public, commercial or not‐for‐profit sectors.

## CONFLICT OF INTEREST STATEMENT

Briony Dow is a member of the editorial board for the Australasian Journal on Ageing. No other conflicts of interest have been disclosed.

## ETHICS STATEMENT

As a Review Article (peer‐reviewed), an Ethics Statement is not applicable.

## Supporting information


Appendices S1–S5

## Data Availability

Data may be available from the corresponding author upon request.
