# Supplementary material for: Understanding factors influencing personal care workers' intentions to stay: A systematic integrative review
Source: Australas J Ageing. 2025 Mar 17;44(1):e70017. doi: 10.1111/ajag.70017 (PMC11912523; doi:10.1111/ajag.70017)
Supplement: Supplementary file 1 — Appendices S1–S5 [file AJAG-44-0-s001.docx]

**Appendix S1:** Search strategy

**Business Source Compete (EBSCO)**

(Date limited 1997 - 18 March 2024)

| **Number** | **Query** |
| --- | --- |
| S59 | S16 AND S48 AND S58 |
| S58 | S49 OR S50 OR S51 OR S52 OR S53 OR S54 OR S55 OR S56 OR S57 |
| S57 | DE "NURSING home chains" |
| S56 | DE "NURSING care facilities" |
| S55 | DE "RESIDENTIAL care" |
| S54 | TI “assisted living facilit*” OR AB “assisted living facilit*” |
| S53 | TI “home* for the aged” OR AB “home* for the aged” |
| S52 | TI ("long term care" OR "long-term care") OR AB ("long term care" OR "long-term care") |
| S51 | TI “nurs* home*” OR AB “nurs* home*” |
| S50 | TI “aged care” OR AB “aged care” |
| S49 | TI residential OR AB residential |
| S48 | S17 OR S18 OR S19 OR S20 OR S21 OR S22 OR S23 OR S24 OR S25 OR S26 OR S27 OR S28 OR S29 OR S30 OR S31 OR S32 OR S33 OR S34 OR S35 OR S36 OR S37 OR S38 OR S39 OR S40 OR S41 OR S42 OR S43 OR S44 OR S45 OR S46 OR S47 |
| S47 | DE "NURSING home employees" |
| S46 | TI "health personnel unlicensed" OR AB "health personnel unlicensed" |
| S45 | TI "blue collar worker*" OR AB "blue collar worker*" |
| S44 | TI “aged care” OR AB “aged care” |
| S43 | TI “elder* care worker*” OR AB “elder* care worker*” |
| S42 | TI “support worker*” OR AB “support worker*” |
| S41 | TI “residential care worker*” OR AB “residential care worker*” |
| S40 | TI “residential worker*” OR AB “residential worker*” |
| S39 | TI “residential support worker*” OR AB “residential support worker*” |
| S38 | TI “personal care assistant*” OR AB “personal care assistant*” |
| S37 | TI “personal assistant*” OR AB “personal assistant*” |
| S36 | TI aid* OR AB aid* |
| S35 | TI caregiver* OR AB caregiver* |
| S34 | TI “care assistant*” OR AB “care assistant*” |
| S33 | TI “care worker*” OR AB “care worker*” |
| S32 | TI “care aid*” OR AB “care aid*” |
| S31 | TI “unlicensed nurs*” OR AB “unlicensed nurs*” |
| S30 | TI “nurs* assistant*” OR AB “nurs* assistant*” |
| S29 | TI “nurs* support*” OR AB “nurs* support*” |
| S28 | TI “nurs* aid*” OR AB “nurs* aid*” |
| S27 | TI frontline OR AB frontline |
| S26 | TI “front line” OR AB “front line” OR frontline |
| S25 | TI directcare OR AB directcare |
| S24 | TI “direct care” AB “direct care” |
| S23 | TI “personal care attendant*” OR AB “personal care attendant*” |
| S22 | TI “personal care worker*” OR AB “personal care worker*” |
| S21 | TI workforce* OR AB workforce* |
| S20 | TI employe* OR AB employe* |
| S19 | TI staff* OR AB staff* |
| S18 | TI personnel* OR AB personnel* |
| S17 | TI worker* OR AB worker* |
| S16 | S1 OR S2 OR S3 OR S4 OR S5 OR S6 OR S7 OR S8 OR S9 OR S10 OR S11 OR S12 OR S13 OR S14 OR S15 |
| S15 | DE "RESIGNATION of employees" |
| S14 | DE "LABOR turnover" |
| S13 | TI stay* OR AB stay* |
| S12 | TI quit* OR AB quit* |
| S11 | TI leave* OR AB leave* |
| S10 | TI attrition OR AB attrition |
| S9 | TI retention OR AB retention |
| S8 | TI shortage OR AB shortage |
| S7 | TI voluntary OR AB voluntary |
| S6 | TI “turnover* rate*” OR AB “turnover* rate*” |
| S5 | TI “actual turnover*” OR AB “actual turnover*” |
| S4 | TI turnover* OR AB turnover* |
| S3 | TI “intention* to stay” OR AB “intention* to stay” |
| S2 | TI “turnover intention*” OR AB “turnover intention*” |
| S1 | TI “intention* to leave” OR AB “intention* to leave” |

**CINAHL Compete (EBSCO)**

(Date limited 1997 - 18 March 2024)

| **Number** | **Query** |
| --- | --- |
| S54 | S17 AND S47 AND S53 |
| S53 | S48 OR S49 OR S50 OR S51 OR S52 |
| S52 | TI “assisted living facilit*” OR AB “assisted living facilit*” |
| S51 | TI “home* for the aged” OR AB “home* for the aged” |
| S50 | TI ("long term care" OR "long-term care") OR AB ("long term care" OR "long-term care") |
| S49 | TI “nurs* home*” OR AB “nurs* home*” |
| S48 | TI “residential aged care” OR AB “residential aged care” |
| S47 | S18 OR S19 OR S20 OR S21 OR S22 OR S23 OR S24 OR S25 OR S26 OR S27 OR S28 OR S29 OR S30 OR S31 OR S32 OR S33 OR S34 OR S35 OR S36 OR S37 OR S38 OR S39 OR S40 OR S41 OR S42 OR S43 OR S44 OR S45 OR S46 |
| S46 | MM "Frontline Employees" |
| S45 | TI "health personnel unlicensed" OR AB "health personnel unlicensed" |
| S44 | TI "blue collar worker*" OR AB "blue collar worker*" |
| S43 | TI “aged care” OR AB “aged care” |
| S42 | TI “elder* care worker*” OR AB “elder* care worker*” |
| S41 | TI “support worker*” OR AB “support worker*” |
| S40 | TI “residential care worker*” OR AB “residential care worker*” |
| S39 | TI “residential worker*” OR AB “residential worker*” |
| S38 | TI “residential support worker*” OR AB “residential support worker*” |
| S37 | TI “personal care assistant*” OR AB “personal care assistant*” |
| S36 | TI “personal assistant*” OR AB “personal assistant*” |
| S35 | TI aide* OR AB aide* |
| S34 | TI caregiver* OR AB caregiver* |
| S33 | TI “care assistant*” OR AB “care assistant*” |
| S32 | TI “care worker*” OR AB “care worker*” |
| S31 | TI “care aide*” OR AB “care aide*” |
| S30 | TI “unlicensed nurs*” OR AB “unlicensed nurs*” |
| S29 | TI “nurs* assistant*” OR AB “nurs* assistant*” |
| S28 | TI “nurs* support*” OR AB “nurs* support*” |
| S27 | TI “nurs* aide*” OR AB “nurs* aide*” |
| S26 | TI frontline OR AB frontline |
| S25 | TI “direct care” OR AB “direct care” |
| S24 | TI “personal care attendant*” OR AB “personal care attendant*” |
| S23 | TI “personal care worker*” OR AB “personal care worker*” |
| S22 | TI workforce* OR AB workforce* |
| S21 | TI employe* OR AB employe* |
| S20 | TI staff*” OR AB staff* |
| S19 | TI personnel* OR AB personnel* |
| S18 | TI worker* OR AB worker* |
| S17 | S1 OR S2 OR S3 OR S4 OR S5 OR S6 OR S7 OR S8 OR S9 OR S10 OR S11 OR S12 OR S13 OR S14 OR S15 OR S16 |
| S16 | MM "Employee Turnover" |
| S15 | MM "Behavioral Intention" |
| S14 | MM "Planned Behavior" |
| S13 | TI stay* OR AB stay* |
| S12 | TI quit* OR AB quit* |
| S11 | TI leave* OR AB leave* |
| S10 | TI attrition* OR AB attrition* |
| S9 | TI retention* OR AB retention* |
| S8 | TI shortage* OR AB shortage* |
| S7 | TI “voluntary turnover” OR AB “voluntary turnover” |
| S6 | TI “turnover rate*” OR AB “turnover rate*” |
| S5 | TI “actual turnover” OR AB “actual turnover” |
| S4 | TI turnover OR AB turnover* |
| S3 | TI “intention* to stay” OR AB “intention* to stay” |
| S2 | TI “turnover intention*” OR AB “turnover intention*” |
| S1 | TI “intention* to leave” OR AB “intention* to leave” |

**Medline Compete (EBSCO)**

(Date limited 1997 - 18 March 2024)

| **Number** | **Query** |
| --- | --- |
| S55 | S15 AND S46 AND S54 |
| S54 | S47 OR S48 OR S49 OR S50 OR S51 OR S52 OR S53 |
| S53 | MM "Residential Facilities+" |
| S52 | TI “assisted living facilit*” OR AB “assisted living facilit*” |
| S51 | TI “home* for the aged” OR AB “home* for the aged” |
| S50 | TI ("long term care" OR "long-term care") OR AB ("long term care" OR "long-term care") |
| S49 | TI “nurs* home*” OR AB “nurs* home*” |
| S48 | TI “aged care” OR AB “aged care” |
| S47 | TI residential OR AB residential |
| S46 | S16 OR S17 OR S18 OR S19 OR S20 OR S21 OR S22 OR S23 OR S24 OR S25 OR S26 OR S27 OR S28 OR S29 OR S30 OR S31 OR S32 OR S33 OR S34 OR S35 OR S36 OR S37 OR S38 OR S39 OR S40 OR S41 OR S42 OR S43 OR S44 OR S45 |
| S45 | TI "health personnel, unlicensed" OR AB "health personnel, unlicensed" |
| S44 | TI "blue collar worker*" OR AB "blue collar worker*" |
| S43 | TI “aged care” OR AB “aged care” |
| S42 | TI “elder* care worker*” OR AB “elder* care worker*” |
| S41 | TI “residential care worker*” OR AB “residential care worker*” |
| S40 | TI “residential care worker*” OR AB “residential care worker*” |
| S39 | TI “residential worker*” OR AB “residential worker*” |
| S38 | TI “residential support worker*” OR AB “residential support worker*” |
| S37 | TI “personal care assistant*” OR AB “personal care assistant*” |
| S36 | TI “personal assistant*” OR AB “personal assistant*” |
| S35 | TI aid* OR AB aid* |
| S34 | TI caregiver* OR AB caregiver* |
| S33 | TI “care assistant*” OR AB “care assistant*” |
| S32 | TI “care worker*” OR AB “care worker*” |
| S31 | TI “care aid*” OR AB “care aid*” |
| S30 | TI “unlicensed nurs*” OR AB “unlicensed nurs*” |
| S29 | TI “nurs* assistant*” OR AB “nurs* assistant*” |
| S28 | TI “nurs* support*” OR AB “nurs* support*” |
| S27 | TI “nurs* aid*” OR AB “nurs* aid*” |
| S26 | TI frontline OR AB frontline |
| S25 | TI “front line” OR AB “front line” |
| S24 | TI directcare OR AB directcare |
| S23 | TI “direct care” OR AB “direct care” |
| S22 | TI “personal care attendant*” OR AB “personal care attendant*” |
| S21 | TI “personal care worker*” OR AB “personal care worker*” |
| S20 | TI workforce* OR AB workforce* |
| S19 | TI employe* OR AB employe* |
| S18 | TI staff* OR AB staff* |
| S17 | TI personnel* OR AB personnel* |
| S16 | TI worker* OR AB worker* |
| S15 | S1 OR S2 OR S3 OR S4 OR S5 OR S6 OR S7 OR S8 OR S9 OR S10 OR S11 OR S12 OR S13 OR S14 |
| S14 | MM "Personnel Turnover" |
| S13 | TI stay* OR AB stay* |
| S12 | TI quit* OR AB quit* |
| S11 | TI leave* OR AB leave* |
| S10 | TI attrition OR AB attrition |
| S9 | TI retention OR AB retention |
| S8 | TI shortage OR AB shortage |
| S7 | TI voluntary OR AB voluntary |
| S6 | TI “turnover* rate*” OR AB “turnover* rate*” |
| S5 | TI “actual turnover*” OR AB “actual turnover*” |
| S4 | TI turnover* OR AB turnover* |
| S3 | TI “intention* to stay” OR AB “intention* to stay” |
| S2 | TI “turnover intention*” OR AB “turnover intention*” |
| S1 | TI “intention* to leave” OR AB “intention* to leave” |

**APA PsycInfo (EBSCO)**

(Date limited 1997 - 18 March 2024)

| **Number** | **Query** |
| --- | --- |
| S54 | S17 AND S47 AND S53 |
| S53 | S48 OR S49 OR S50 OR S51 OR S52 |
| S52 | TI “assisted living facilit*” OR AB “assisted living facilit*” |
| S51 | TI “home* for the aged” OR AB “home* for the aged” |
| S50 | TI ("long term care" OR "long-term care") OR AB ("long term care" OR "long-term care") |
| S49 | TI “nurs* home*” OR AB “nurs* home*” |
| S48 | TI “residential aged care” OR AB “residential aged care” |
| S47 | S18 OR S19 OR S20 OR S21 OR S22 OR S23 OR S24 OR S25 OR S26 OR S27 OR S28 OR S29 OR S30 OR S31 OR S32 OR S33 OR S34 OR S35 OR S36 OR S37 OR S38 OR S39 OR S40 OR S41 OR S42 OR S43 OR S44 OR S45 OR S46 |
| S46 | MM "Frontline Employees" |
| S45 | TI "health personnel unlicensed" OR AB "health personnel unlicensed" |
| S44 | TI "blue collar worker*" OR AB "blue collar worker*" |
| S43 | TI “aged care” OR AB “aged care” |
| S42 | TI “elder* care worker*” OR AB “elder* care worker*” |
| S41 | TI “support worker*” OR AB “support worker*” |
| S40 | TI “residential care worker*” OR AB “residential care worker*” |
| S39 | TI “residential worker*” OR AB “residential worker*” |
| S38 | TI “residential support worker*” OR AB “residential support worker*” |
| S37 | TI “personal care assistant*” OR AB “personal care assistant*” |
| S36 | TI “personal assistant*” OR AB “personal assistant*” |
| S35 | TI aide* OR AB aide* |
| S34 | TI caregiver* OR AB caregiver* |
| S33 | TI “care assistant*” OR AB “care assistant*” |
| S32 | TI “care worker*” OR AB “care worker*” |
| S31 | TI “care aide*” OR AB “care aide*” |
| S30 | TI “unlicensed nurs*” OR AB “unlicensed nurs*” |
| S29 | TI “nurs* assistant*” OR AB “nurs* assistant*” |
| S28 | TI “nurs* support*” OR AB “nurs* support*” |
| S27 | TI “nurs* aide*” OR AB “nurs* aide*” |
| S26 | TI frontline OR AB frontline |
| S25 | TI “direct care” OR AB “direct care” |
| S24 | TI “personal care attendant*” OR AB “personal care attendant*” |
| S23 | TI “personal care worker*” OR AB “personal care worker*” |
| S22 | TI workforce* OR AB workforce* |
| S21 | TI employe* OR AB employe* |
| S20 | TI staff*” OR AB staff* |
| S19 | TI personnel* OR AB personnel* |
| S18 | TI worker* OR AB worker* |
| S17 | S1 OR S2 OR S3 OR S4 OR S5 OR S6 OR S7 OR S8 OR S9 OR S10 OR S11 OR S12 OR S13 OR S14 OR S15 OR S16 |
| S16 | MM "Employee Turnover" |
| S15 | MM "Behavioral Intention" |
| S14 | MM "Planned Behavior" |
| S13 | TI stay* OR AB stay* |
| S12 | TI quit* OR AB quit* |
| S11 | TI leave* OR AB leave* |
| S10 | TI attrition* OR AB attrition* |
| S9 | TI retention* OR AB retention* |
| S8 | TI shortage* OR AB shortage* |
| S7 | TI “voluntary turnover” OR AB “voluntary turnover” |
| S6 | TI “turnover rate*” OR AB “turnover rate*” |
| S5 | TI “actual turnover” OR AB “actual turnover” |
| S4 | TI turnover OR AB turnover* |
| S3 | TI “intention* to stay” OR AB “intention* to stay” |
| S2 | TI “turnover intention*” OR AB “turnover intention*” |
| S1 | TI “intention* to leave” OR AB “intention* to leave” |

**Embase (Embase.com)**

(Date limited 1997 - 18 March 2024)

| **Number** | **Query** |
| --- | --- |
| #54 | #52 AND 'article'/it AND [english]/lim |
| #53 | #52 AND 'article'/it |
| #52 | #14 AND #43 AND #51 |
| #51 | #44 OR #45 OR #46 OR #47 OR #48 OR #49 OR #50 |
| #50 | 'assisted living facilit*':ab,ti AND [embase]/lim |
| #49 | 'home* for the aged':ab,ti AND [embase]/lim |
| #48 | 'long-term care':ab,ti AND [embase]/lim |
| #47 | 'long term care':ab,ti AND [embase]/lim |
| #46 | 'nurs* home*':ab,ti AND [embase]/lim |
| #45 | 'aged care':ab,ti AND [embase]/lim |
| #44 | 'residential':ab,ti AND [embase]/lim |
| #43 | #15 OR #16 OR #17 OR #18 OR #19 OR #20 OR #21 OR #22 OR #23 OR #24 OR #25 OR #26 OR #27 OR #28 OR #29 OR #30 OR #31 OR #32 OR #33 OR #34 OR #35 OR #36 OR #37 OR #38 OR #39 OR #40 OR #41 OR #42 |
| #42 | 'health personnel, unlicensed':ab,ti AND [embase]/lim |
| #41 | 'blue collar worker*':ab,ti AND [embase]/lim |
| #40 | 'aged care':ab,ti AND [embase]/lim |
| #39 | 'elder* care worker*':ab,ti AND [embase]/lim |
| #38 | 'support worker*':ab,ti AND [embase]/lim |
| #37 | 'residential care worker*':ab,ti AND [embase]/lim |
| #36 | 'residential worker*':ab,ti AND [embase]/lim |
| #35 | 'residential support worker*':ab,ti AND [embase]/lim |
| #34 | 'personal care assistant*':ab,ti AND [embase]/lim |
| #33 | 'personal assistant*':ab,ti AND [embase]/lim |
| #32 | aid*:ab,ti AND [embase]/lim |
| #31 | 'caregiver*':ab,ti AND [embase]/lim |
| #30 | 'care assistant*':ab,ti AND [embase]/lim |
| #29 | 'care worker*':ab,ti AND [embase]/lim |
| #28 | 'care aid*':ab,ti AND [embase]/lim |
| #27 | 'unlicensed nurs*':ab,ti AND [embase]/lim |
| #26 | 'nurs* assistant*':ab,ti AND [embase]/lim |
| #25 | 'nurs* support*':ab,ti AND [embase]/lim |
| #24 | nurs*:ab,ti AND [embase]/lim |
| #23 | 'front line worker*':ab,ti AND [embase]/lim |
| #22 | 'direct care worker*':ab,ti AND [embase]/lim |
| #21 | 'personal care attendant*':ab,ti AND [embase]/lim |
| #20 | 'personal care worker*':ab,ti AND [embase]/lim |
| #19 | workforce*:ab,ti AND [embase]/lim |
| #18 | employe*:ab,ti AND [embase]/lim |
| #17 | staff*:ab,ti AND [embase]/lim |
| #16 | personnel*:ab,ti AND [embase]/lim |
| #15 | worker*:ab,ti AND [embase]/lim |
| #14 | #1 OR #2 OR #3 OR #4 OR #5 OR #6 OR #7 OR #8 OR #9 OR #10 OR #11 OR #12 OR #13 |
| #13 | stay:ab,ti AND [embase]/lim |
| #12 | quit:ab,ti AND [embase]/lim |
| #11 | leave:ab,ti AND [embase]/lim |
| #10 | attrition:ab,ti AND [embase]/lim |
| #9 | retention:ab,ti AND [embase]/lim |
| #8 | shortage:ab,ti AND [embase]/lim |
| #7 | voluntary:ab,ti AND [embase]/lim |
| #6 | 'turnover rate':ab,ti AND [embase]/lim |
| #5 | 'actual turnover*':ab,ti AND [embase]/lim |
| #4 | turnover*:ab,ti AND [embase]/lim |
| #3 | 'intention* to stay':ab,ti AND [embase]/lim |
| #2 | 'turnover intention*':ab,ti AND [embase]/lim |
| #1 | 'intention* to leave':ab,ti AND [embase]/lim |

**Appendix S2:** Quantitative summary of included studies

| **Author**  **(Year)**  **Country** | **Aim/objective** | **Study design** | **Setting, participant sample size, response rate & characteristics** | **Data collection methods** | **Data analysis** | **Results (variables demonstrating statistical significance were extracted and reported from the highest level of analysis)** | **Limitations** |
| --- | --- | --- | --- | --- | --- | --- | --- |
| Bishop et al^20^ (2008), USA | To investigate (a) whether CNAs are more committed to nursing home jobs when they perceive their jobs as enhanced (greater autonomy, use of knowledge, teamwork), and (b) whether CNA job commitment affects resident satisfaction. | Cross-sectional, surveys | Setting: 18 purposively selected Massachusetts NHs  Participation rate: 83% (15/18) NHs  Participant sample: n =  255 CNAs, 105 residents  Response rate: 95% CNAs (255/267), 85% residents (105/123)  Participant characteristics  Gender: 87 % female  Age (years): 39% <35, 31%, >45 31%  Tenure: 24% <1 year in NH  Race: 25% white  Employment intentions (ITL): 55% No | Workplace relationships, job satisfaction, and resident care: 82 items – based on interview data.  Resident satisfaction with staff relationships: 38 questions adapted from University of Minnesota instrument.  Resident quality of life: adapted Short Quality of Life index, 14 items. | Descriptive statistics.  Logistic regression to examine the relationship between personal characteristics, satisfaction with tangible job rewards, and elements of job design on CNAs’ ITS in current jobs.  General linear model estimated the effect of job commitment on residents’ satisfaction with their relationships with staff. | In the logistic regression analysis employing a parsimonious model, Panel 2, basic supervision significantly improved the model, while job design enhancements in Panels 3-5 did not add significantly to explanatory power. ITL revealed a positive association with employee benefits (0.580, *p* < .1), career advancement (0.793, *p* < .01), basic supervision (1.337, *p* < .01), and the personal characteristic of age, particularly those older than 45 years (0.868, *p* < .01). | Unreported by Bishop et al. Authors interpretations of limitations: limited sample size and generalisability, reliance on self-reported data, and potential CNA confounding factors. A cross-sectional design hindering causal inference, and a focus on specific variables. |
| Chang et al^40^ (2021), Taiwan | To investigate the influences of nursing assistants' job competency on their intrinsic and extrinsic satisfaction and their ITS in the profession of LTC institutions. | Cross-sectional questionnaire | Setting: unreported sampling strategy, 26 NHs and 15 elderly welfare institutes that were certificated in Taichung city, Taiwan  Participation rate: unreported  Participant sample: n = 333 NAs  Response rate: 87% (333/383)  Participant characteristics  Gender: 84.4% female  Age (in years): 22% 30-39 21%, 40-49, 28%, 50-59, 13% >60  Tenure (years): 25.8% 5-10, 22%, >10  Race: unreported  Employment intentions: unreported | Job competency: adapted from Long-term care, supports, and services competency model, 5 domains, 21 items, using 5-point Likert scale.  Job satisfaction: Minnesota Satisfaction Questionnaire, 20 items, using 5-point Likert scale.  ITS: Milliman, Gatling, and Kim, two items using 5-point Likert scale | Partial least squares (PLS) analysis to develop predictive models for latent variables.  Bootstrap resampling method to draw 5000 samples for parameter calculations and inference estimations.  PLS to estimate path relationships between job competency, intrinsic and extrinsic job satisfaction, and intention to stay among nursing assistants. | A direct significant, positive relationships between NAs' intrinsic satisfaction (0.237, 95% CI: 0.016, 0.434, *p* < .05), and extrinsic satisfaction (0.321, 95% CI: 0.135, 0.497, *p* < .01) was found.  A positive mediating relationship suggested that job competency indirectly impacts ITS through the mediating effects of intrinsic (0.082, 95% CI: 0.009, 0.158, *p* < .05) and extrinsic job satisfaction (point estimate = 0.157, 95% CI: 0.065, 0.256, *p* < .001).  Additionally, job competency was found to indirectly impact ITS through medication of extrinsic and intrinsic satisfaction (0.072, 95% CI: 0.006, 0.150, *p* < .05). | Cross-sectional design and despite use of PLS for causal model analysis, not possible to draw causal conclusions, the inadequate number of participants, and only two mediating factors looked at the mediating effects on job competency and ITS. |
| Dhakal et al^31^ (2020), Australia | To explore the attraction and retention of ACAs and to identify specific 'push-and-pull' factors influencing the intention of ACAs to stay in their current job or leave for other occupations. | Cross-sectional survey | Setting: 9 purposively selected RAC settings in Western Australia, Australia  Participation rate: unreported  Participant sample: n = 79 ACAs  Response rate: 20% (79/391)  Participant characteristics  Gender: 89% female  Age (years): 46% <39, 54% >40  Tenure (years): 47% <4, 53%, >5 in aged care sector  Race: unreported  Employment intentions: 40% ITL current employer, 22% ITL aged care sector | 20 questions covering the four areas: (1) the nature of work and the working environment (2) the importance of personal qualities for the job (3) respondent's ITS/ITL over the next 12 months (4) perceived requisite attributes of ACAs. | Two by-two cross tabulation to explore issues about attraction/retention of ACAs.  χ^2^ test determine statistically significant associations. | ITS and continue working over the next 12 months were positively associated with current employment status (permanent or temporary) (χ2 (n = 79) = 14.8, *df* = 1, *p* < .001), education attainment (degree/diploma or higher) (χ2 (n = 70) = 5.6, *df* = 1, *p* < .05), age of 40 years and above (χ2 (n = 78) = 17.4, *df* = 1, *p* < .001), employees who did not speak a language other than English at home (χ2 (n = 79) = 7.5, *df* 1, *p* < .05), and employment in a non-metropolitan area (χ2 (n = 78) = 16.0, *df* = 1, *p* < .001). | Low response rate impeded the generalisability of the findings. |
| Dill et al^25^ (2013), USA | To examine the relationship between job satisfaction, ITS, and retention among low-wage health care workers, specifically focusing on NAs in NHs. | Cross-sectional survey | Setting: 18 purposively and convenience selected NHs in a southern USA state  Participation rate: not reported  Participant sample: n = 315 NAs  Response rate: 95% (n = 449)  Participant characteristics  Gender: unreported  Age: unreported  Tenure: unreported  Race: unreported  Employment intentions: unreported | ITS: three items, assessing ITS in current position, occupation as NA, field of long-term care, using 4-point scales and YES for stay in field 3-years from now.  Job satisfaction, four items.  Perceived job characteristics: supervisory support, eight items workload, three items financial  rewards, three items career rewards, three items.  Quality of care: perceived quality of care 16 items, quality of coworkers nine items and team care four items.  Contingency factors: being a primary breadwinner, being a single mother, receiving public assistance, and having health insurance. | Logistic regression models were used to analyse retention and ITS in the field of LTC.  Ordered logit models were used to analyse the ITS in one's job and ITS in the occupation of NAs. | Model 1 showed that ITS in current position for the near future was significantly associated with job satisfaction. (coef. = 0.65, *p* ≤ .001) and public assistance (coef. = 0.61, *p* ≤ .05). Job quality characteristics and contingency factors on ITS among NAs revealed that supervisor support (coef. = -0.70, *p* ≤ .01), financial rewards (coef. = 0.46, *p* ≤ .05), career rewards (coef. = 0.58, *p* ≤ .05), perceived quality of care (coef. = 1.03, *p* ≤ .05), being the household breadwinner (coef. = 0.46, *p* ≤ .05), and receiving public assistance (coef. = 0.75, *p* ≤ .01) were significantly associated with NAs ITS in their current positions for the near future. | Proxies were employed, hence specific processes for employee turnover could not be determined. Unable to differentiate between voluntary and involuntary turnover. High level of missing data potentially reduced independent variables’ variability and added to many of the models having low levels of described variance. |

**Abbreviations**: ACA: Aged care assistant; CNA: Certified nursing assistant; Coef.: Coefficient; ITL: Intention to leave: ITS: Intention to stay; NAs: Nursing assistants; LTC: Long-term care; NH: Nursing home; RAC: Residential aged care.

**Notes:** Sample size = the respective studies sample for analysis.

**Appendix S3:** Statistically non-significant results of included quantitative studies

| **Reference** | **Results** |
| --- | --- |
| Bishop et al^20^ (2008), USA | Regression analysis, Panel 2 basic supervision, revealed wages (coefficient of 0.496) was the only variable statistically not significant concerning employee’s intentions to stay. |
| Chang et al^40^ (2021), Taiwan | The study did not yield any statistically not significant results concerning employee’s intentions to stay. |
| Dhakal et al^31^ (2020), Australia | The χ^2^ analysis revealed that the following variables were found not statistically significant, gender (χ^2^ (n = 79) = 2.8, *df* = 1, *p* < .24) and years working in the aged care sector (χ^2^ (n = 78) = 3.2, *df* = 1, *p* < .1). |
| Dill et al^25^ (2013), USA | Logistic regression, Model 1, with job quality characteristics as independent variables, revealed that several variables were statistically not significant concerning employee’s intentions to stay. These included workload (Coefficient = -0.37, SE = 0.20), quality of coworkers (Coefficient = 0.19, SE = 0.35), teamwork (Coefficient = -0.41, SE = 0.33), single mother (Coefficient = -0.56, SE = 0.31), and receiving health insurance (Coefficient = -0.19, SE = 0.20). |
| Yeatts & Cready^42^ (2007), USA | The study did not yield any statistically not significant results regarding employee’s intentions to stay. |

**Appendix S4:** Qualitative summary of included studies

| **Author**  **(Year)**  **Country** | **Aim/objective** | **Study design** | **Setting**  **Participant sample size, response rate & characteristics** | **Data collection methods** | **Data analysis** | **Results** | **Limitations** | |
| --- | --- | --- | --- | --- | --- | --- | --- | --- |
| Gao et al^41^ (2015), Australia | To understand individual DCWs' perceptions of the rewards and difficulties of RAC work, how these were related to their employment intentions, and how these varied between nurses and NAs, and the cultural diversity of workers. | Individual semi-structured interviews | Setting: 1 non-profit RAC setting in Queensland, Australia  Participant rate: unreported  Participant sample: n = 16 (10 NAs, 6 nurses), non-purposive sampling  Response rate: 84% (16/19)  Participant characteristics of NAs:  Gender: 80% female  Age (years): 30% 25-34, 20% 35-44, 10% 45-54, 40% 55-65  Tenure (year): 30% < 1, 40% 1-5 30% > 6 at the RAC  Race: 60% born overseas  Employment intentions: 70% stay, 30% leave | Domains in Interview Guide:   - rewarding features - difficult aspects - attraction - retention - additional question | Thematic analysis | DCWs' employment intentions were found to be related to their perceptions and management of rewards and difficulties of care work in four major domains: (1) nature of care work, (2) employment characteristics, (3) organisational resources and (4) meaning of care work.  NAs' employment intentions were strongly linked to their evaluation and handling of the challenges and rewards of care work. | Single not-for-profit RAC setting.  Potential bias due to facility being well regarded by residents/family and link between supportive management and DCW high job satisfaction. Only perceptions of DCWs captured exploring employment intentions at one point in time.  Small-scale size of study. | |
| Yeatts & Cready^42^ (2007), USA | To evaluate the effects of empowered work teams, specifically designed to empower CNAs, within the LTC setting. | Mixed methods, observations, empowered work team weekly summaries, and nurse management weekly written responses | Setting: 10 purposefully selected NHs (5 experimental groups and 5 control groups) in the north Texas region, America  Participation rate: 27.7% (5/18) experimental (E); control (C) unreported    Participant sample: n = 314 to 353 CNAs  Response rate:  CNAs E NH = 84%  CNAs C NH = 92%  Participant characteristics of CNAs  Gender: E 83% female, C 92% female  Age: E mean 36.2 years, C mean 37.5 years  Tenure: E mean 42 months at NH, C mean 36.7 months  Race: E 52% non-Hispanic white, 32% non-Hispanic black, 16% Hispanic or other, C 42% non-Hispanic white, 40% non-Hispanic black, 18% Hispanic or other  Employment intentions: unreported | Observations of over 270 CNA team meetings and examination of weekly team-meeting summaries provided by the CNA-empowered work teams to nurse management. | Qualitative data reviewed separately for each proposition being tested. The researchers looked for trends that both supported and refuted the propositions, involving involved looking for patterns, themes, and insights within the qualitative data to draw conclusions about the effects of empowered work teams on various aspects of the NH. | From the observation data gathered, CNAs’ employment intentions were found to be related to empowered CNA teams. | Generalisability of the findings was limited due to the selected study design of quasi-experimental conducted in a single region. Potential for selection bias as NHs voluntarily participated.  CNAs, nurses and family members were not randomised to NHs so potential for further differences between groups.  Data analysis was only performed on those who attended both pretest and post-test 16 months later. Interpreting the turnover rate requires caution, with the comparison group having an extra month for CNAs to resign. The absence of pre-study employment data precludes a definitive determination regarding the exclusive influence of empowered work teams. |  |

**Abbreviations:** C: Control; CNA: Certified nursing assistant; DCW: Direct care worker; E: Experimental; LTC: Long-term care; NH: Nursing home; RAC: Residential aged care.

**Notes:** The findings from the qualitative study by Gao et al (2015), were extracted from the study prior to be thematically analysed.

Sample size = the respective studies sample for analysis.

**Appendix S5:** Mixed Methods Appraisal Tool (MMAT)

| **Qualitative study design** | **Gao et al^41^ (2015), Australia** |  |  |  |
| --- | --- | --- | --- | --- |
| - 1. Is the qualitative approach appropriate to answer the research question?   2. Are the qualitative data collection methods adequate to address the research question?   3. Are the findings adequately derived from the data?   4. Is the interpretation of results sufficiently substantiated by data?   5. Is there coherence between qualitative data sources, collection, analysis and interpretation?   **Total score** | 1  1  1  1  1  **5** |  |  |  |
| **Quantitative descriptive study design** | **Bishop et al^20^ (2008), USA** | **Chang et al^40^ (2021), Taiwan** | **Dhakal et al^31^ (2020), Australia** | **Dill et al^25^ (2013), USA** |
| 4.1. Is the sampling strategy relevant to address the research question?  4.2. Is the sample representative of the target population?  4.3. Are the measurements appropriate?  4.4. Is the risk of non-response bias low?  4.5. Is the statistical analysis appropriate to answer the research question?  **Total score** | 1  1  1  1  1  **5** | 1  1  1  1  1  **5** | 1  1  1  0  0  **3** | 1  1  1  1  1  **5** |
| **Mixed methods study design** | **Yeatts & Cready^42^ (2007), USA** |  |  |  |
| 5.1. Is there an adequate rationale for using a mixed methods design to address the research question?  5.2. Are the different components of the study effectively integrated to answer the research question?  5.3. Are the outputs of the integration of qualitative and quantitative components adequately interpreted?  5.4. Are divergences and inconsistencies between quantitative and qualitative results adequately addressed?  5.5. Do the different components of the study adhere to the quality criteria of each tradition of the methods involved?  **Total score** | 0  1  1  1  0  **3** |  |  |  |
|  |  |  |  |  |

**Notes:** 1 = a score of ‘yes’; 0 = a score of ‘no’ or ‘can’t tell’.

Mixed methods studies were given the score of their lowest scoring criterion, as recommended by MMAT.
